# Identification of extracellular siderophores and a related peptide from the endophytic fungus *Epichloë festucae* in culture and endophyte-infected *Lolium perenne*

**DOI:** 10.1016/j.phytochem.2011.11.020

**Published:** 2012-03

**Authors:** Albert Koulman, T. Verne Lee, Karl Fraser, Linda Johnson, Vickery Arcus, J. Shaun Lott, Susanne Rasmussen, Geoffrey Lane

**Affiliations:** a Lipid Profiling and Signaling Group, MRC HNR, Elsie Widdowson Laboratory, Cambridge, UK; b AgResearch Structural Biology Laboratory, School of Biological Sciences, University of Auckland, Auckland 1142, New Zealand; c AgResearch Limited, Grasslands Research Centre, Palmerston North 4442, New Zealand; d Department of Biological Sciences, University of Waikato, Hamilton 3240, New Zealand

**Keywords:** *Epichloë festucae*, Clavicipitaceae, *Lolium perenne*, Poaceae, Siderophore, Cyclic peptide, LC-MS*^n^*, High resolution MS*^n^* (HRMS*^n^*), NMR

## Abstract

A number of genes encoding non-ribosomal peptide synthetases (NRPSs) have been identified in fungi of *Epichloë*/*Neotyphodium* species, endophytes of Pooid grasses, including *sidN*, putatively encoding a ferrichrome siderophore-synthesizing NRPS. Targeted gene replacement and complementation of *sidN* in *Epichloë festucae* has established that extracellular siderophore epichloënin A is the major product of the SidN enzyme complex (Johnson et al., 2007a). We report here high resolution mass spectrometric fragmentation experiments and NMR analysis of an isolated fraction establishing that epichloënin A is a siderophore of the ferrichrome family, comprising a cyclic sequence of four glycines, a glutamine and three *N*^δ^-*trans*-anhydromevalonyl–*N*^δ^-hydroxyornithine (AMHO) moieties. Epichloënin A is unusual among ferrichrome siderophores in comprising an octapeptide rather than hexapeptide sequence, and in incorporating a glutamine residue. During this investigation we have established that desferrichrome siderophores with pendant *trans*-AMHO groups can be distinguished from those with pendant *cis*-AMHO groups by the characteristic neutral loss of an hydroxyornithine moiety in the MS/MS spectrum. A minor component, epichloënin B, has been characterized as the triglycine variant by mass spectrometry. A peptide characterized by mass spectrometry as the putative deoxygenation product, epichloëamide has been detected together with ferriepichloënin A in guttation fluid from ryegrass (*Lolium perenne*) plants infected with wild-type *E. festucae*, but not in plants infected with the Δ*sidN* mutant strain, and also detected at trace levels in wild-type *E. festucae* fungal culture.

## Graphical abstract

**Graphical abstract:**

Epichloënin A (**1**) has been isolated from fungal cultures together with a minor variant epichloënin B, and ferriepichloënin A and a related peptide epichloëamide have been found in endophyte-infected plants.

**Figure:**
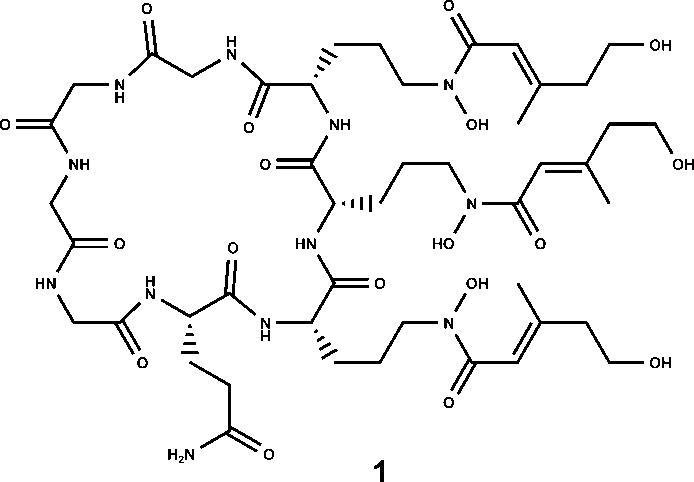

**Highlights:**

► Epichloënin A has been characterized as a ferrichrome octapeptide by HRMS*^n^* and NMR. ► The minor heptapeptide variant epichloënin B has been characterized by HRMS*^n^*. ► Ferriepichloënin A was detected in guttation fluid of endophyte-infected plants. ► The related peptide epichloëamide was also detected in guttation fluid. ► Epichloëamide has been characterized as an octapeptide by HRMS*^n^*.

## 1 Introduction

Investigations of the effects of symbiotic associations of endophytes of *Epichloë*/*Neotyphodium* species (epichloë endophytes; family Clavicipitaceae) with cool season grasses (family Poaceae, subfamily Pooideae) have identified a range of agriculturally important fungal metabolites produced *in planta* which adversely affect grazing livestock and insect herbivores (Bush et al., 1997, Clay and Schardl, 2002). These include pyrrolizidine alkaloids of the loline family (Bush et al., 1993); ergot alkaloids, particularly ergovaline (Lyons et al., 1986); indolediterpenoids, particularly lolitrem B (Gallagher et al., 1984); and the pyrrolopyrazine peramine (Rowan and Gaynor, 1986). For each of these classes evidence has been obtained suggesting they play a role in defending the symbiosis against herbivores whether vertebrate (Siegel and Bush, 1996) or invertebrate (Popay and Bonos, 2005).

Many of the genes and gene complexes involved in the biosynthesis of these fungal metabolites have now been identified and characterized. These include *ltm* genes involved in the synthesis of the neurotoxin lolitrem B and related indolediterpenes in *Neotyphodium lolii* (Young et al., 2005, Young et al., 2006), and *lol* genes involved in the synthesis of insect-toxic loline alkaloids in *Neotyphodium uncinatum* (Schardl et al., 2007, Spiering et al., 2008, Spiering et al., 2005). More pertinent to this study, non-ribosomal peptide synthetase (NRPS) genes and gene clusters have been shown to be involved in the production in epichloë endophytes of the insect feeding deterrent peramine (*perA*) (Tanaka et al., 2005) and the mammalian and insect toxin ergovaline (*LpsA* and *LpsB*) (Fleetwood et al., 2007, Panaccione et al., 2001).

A degenerate PCR based approach to identify NRPS genes from several *Epichloë* and *Neotyphodium* endophytes revealed a number of NRPS additional to those involved in peramine and ergovaline biosynthesis (Johnson et al., 2007b), suggesting that the current understanding of the range of metabolites the fungus contributes to the symbiotum is incomplete. Evidence that additional classes of fungal metabolites to those listed above may be present in endophyte-infected plants has also been provided by metabolomic comparisons of extracts of leaf, pseudostem and seeds of endophyte-infected and endophyte-free perennial ryegrass using direct infusion MS (DIMS) (Cao et al., 2008, Koulman et al., 2007b).

One of the novel NRPS genes, NRPS2, found in all the *Epichloë* and *Neotyphodium* fungal strains examined (Johnson et al., 2007b) and now designated *sidN*, has been cloned from *Epichloë festucae* strain Fl1 (Johnson et al., 2007a). Sequence analysis has shown it has high amino acid sequence similarities and a similar gene structure to previously characterized NRPSs encoding synthetases for ferrichrome siderophores (Bushley et al., 2008, Eisendle et al., 2003, Schwecke et al., 2006). These are typically cyclic hexapeptides comprising three *N*^δ^-acyl-*N*^δ^-hydroxyornithine (hydroxamate) moieties and three proteinogenic amino acids, restricted to glycine, alanine or serine in reported structures (Johnson, 2008, Renshaw et al., 2002). However while the gene evidence suggested the SidN product was a member of the ferrichrome class, its structure could not be directly inferred from the gene sequence data, as the sequence and multiplicity of component amino acids within known siderophores of this class does not correspond to the sequence of modules within the corresponding NRPS gene (Bushley et al., 2008).

Functional analysis of the *sidN* gene has been carried out by construction of Δ*sidN* mutants in *E. festucae* strain Fl1 by targeted gene replacement (Johnson et al., 2007a). Investigations of NRPS genes encoding ferrichrome siderophores have been carried out with fungal cultures (e.g. Yuan et al., 2001) rather than *in planta* as for endophyte NRPSs involved in alkaloid synthesis (Fleetwood et al., 2007, Panaccione et al., 2001, Tanaka et al., 2005). Accordingly, culture supernatants and mycelial extracts of wild-type (WT) *E. festucae*, Δ*sidN* mutant strains and complemented strains grown under iron-depleted conditions were analyzed by LC-MS*^n^*. As reported elsewhere (Johnson et al., 2007a; unpublished data), this revealed the presence of a novel extracellular siderophore, designated as epichloënin A (**1**) (in the desferri-form), and its iron chelate ferriepichloënin A (**1-Fe**) in cultures of WT and complemented strains, but not in Δ*sidN* mutant cultures. Comparative studies of ryegrass (*Lolium perenne*) plants infected with WT and mutant strains have established the siderophore plays an important role in the grass-endophyte symbiosis (Johnson et al., 2007a; unpublished data).

High resolution Fourier transform mass spectrometry (HRFTMS) and MS*^n^* of the desferri-species from WT *E. festucae* Fl1 culture supernatants grown under iron-depleted conditions indicated **1** was a hydroxamate siderophore of molecular formula C_46_H_74_N_12_O_18_ incorporating three *N*^δ^-anhydromevalonyl–*N*^δ^-hydroxyornithine (AMHO) moieties (Lee et al., 2010). Biochemical studies of the third adenylation domain of the SidN NRPS enzyme complex from the related fungus *N. lolii* which produces **1** in culture (Koulman and Lane, unpublished data) established it could bind and activate *cis*-AMHO, but not any of the 20 proteogenic amino acids (Lee et al., 2010). However LC-MS analysis of culture supernatants of Δ*sidN* mutant strains of *E. festucae* established these accumulated *trans*-AMHO rather than *cis*-AMHO as the putative precursor for the siderophore (unpublished data).

The detailed structure of **1** remained to be defined and we describe here its isolation from fungal culture and its structure elucidation by HRMS*^n^* of **1** and ferriepichloënin A (**1-Fe**), and by NMR of **1**. We have extrapolated these findings to elucidate by LC-MS*^n^* the structure of a minor structural variant which we have designated epichloënin B (**2**) which co-occurs with **1** in fungal culture. Guttation fluid which is effectively a regulated waste stream from the plant, provides a clean matrix for the detection of endophyte metabolites *in planta* (Koulman et al., 2007a), and **1-Fe** has been detected in guttation fluid from plants infected with the WT but not the Δ*sidN* mutant strain by LC-MS*^n^* (unpublished data). We here report the discovery in guttation fluid of an associated peptide co-occurring with **1-Fe** which we have designated epichloëamide (**3**) and the elucidation of its structure by LC-MS*^n^*. Compound **3** was subsequently detected at trace levels in extracts of mycelium from WT fungal cultures.

## 2 Results and discussion

### 2.1 Epichloënin A

**Fig. 1:**
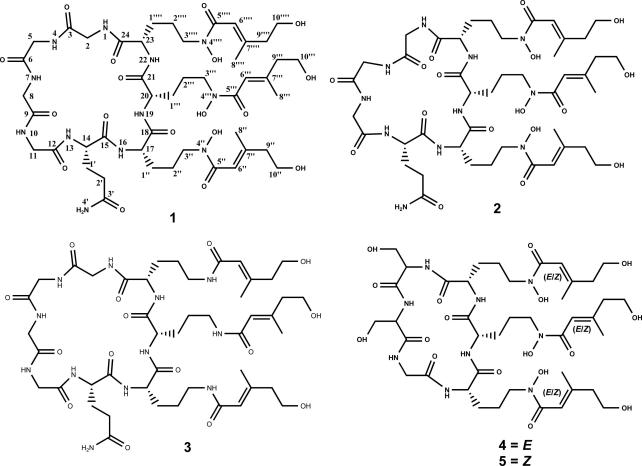
Structures of siderophores and a related peptide from *Epichloë festucae* strain Fl1, and related siderophores.

The evidence from the gene sequence and deletion studies (Johnson et al., 2007a) together with the ligand activation, crystallographic investigation and HRMS*^n^* measurements (Lee et al., 2010) were largely consistent with **1** (Fig. 1) being a siderophore of the ferrichrome class with three pendant *trans-*AMHO moieties, homologous to ferrirubin (**4-Fe**). One finding appeared in conflict with this, namely the observation of neutral loss of an hydroxyornithine residue on HRMS*^n^* of **1** (Lee et al., 2010). This implied the formation of a product ion with retention of all three anhydromevalonyl moieties which suggested an alternative structure containing ester-linked anhydromevalonyl moieties as in coprogen and fusarinine siderophores (Johnson, 2008, Renshaw et al., 2002). In addition, the other component amino acids and other details of the structure remained to be characterized.

Thus further chemical investigation was required to define the structure of **1**. While **1** (*m*/*z* 542 [M+2H]^2+^, *m*/*z* 1084 [M+H]^+^; *λ*_max_ 245 nm) was observed by LC-MS with UV–visible detection as a major component in supernatants of WT *E. festucae* Fl1 cultures grown under iron-depleted conditions, the predominant species in supernatants from iron-replete cultures was the corresponding ferric complex **1-Fe** (*m*/*z* 569 [M+2H]^2+^, 1136 [M+H]^+^), with a visible absorbance (*λ*_max_ 440 nm) characteristic of a ferric-hydroxamate complex. The molecular formula for **1** (above) was confirmed by data from HR positive nanospray MS of **1** and of **1-Fe** in which peaks for both major isotopologues were observed (*m*/*z* 1136.4436 [M(^56^Fe)+H]^+^ (calcd. for C_46_H_72_^56^FeN_12_O_18_^+^, 1136.4432) (100), 1134.4450 [M(^54^Fe)+H]^+^ (calcd. for C_46_H_72_^54^FeN_12_O_18_^+^, 1134.4478) (6)). These data are consistent with the displacement in **1-Fe** of the three hydroxamate protons in **1** by chelated iron.

On HRMS*^n^* of **1-Fe** (*m*/*z* 1136.5) under positive nanospray conditions the major product ions observed corresponded to neutral losses of water and an AMHO moiety, as reported for ferrirubin and ferrirhodin (Mawji et al., 2008), and to the formation of an [Fe-(AMHO)_2_]^+^ complex, as reported for ferrichrome, ferricrocin and ferrichrysin (Essén et al., 2006) consistent with the previous findings for **1** (Lee et al., 2010).

**Fig. 2:**
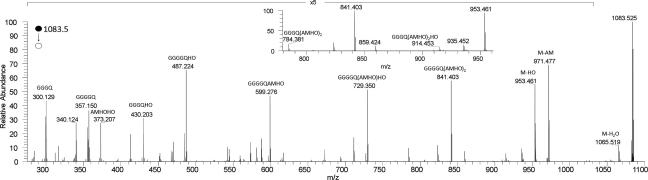
HRMS^2^ spectrum of the protonated cyclic octapeptide **1** ion cyclo-[GGGGQ(AMHO)_3_H]^+^, using Collision Induced Dissociation with different amounts of energy. Product ions are assigned as acylium species ([M+H−H_2_O]^+^) of the peptides shown in the annotation. Inset is a subsection of the spectrum, showing losses of anhydromevalonyl (AM) and AMHO plus glycine.

Further evidence of the component amino acids and their sequence in the peptide was provided by detailed examination of the HRMS*^n^* of **1**. A range of product ions was observed in the HRMS^2^ spectrum of **1** (Fig. 2) and these have been assigned to acylium species of the peptides shown.^1^ Product ions associated with fragmentation of the AMHO moieties dominate the spectrum, including the neutral loss of an hydroxyornithine (HO) residue as previously reported (Lee et al., 2010). Evidence that a glycine residue was located adjacent to an AMHO moiety in **1** was provided by the observation of products corresponding to neutral losses of anhydromevalonyl and a glycine residue and of an AMHO glycine dipeptide residue (Fig. 2, inset).

**Fig. 3:**
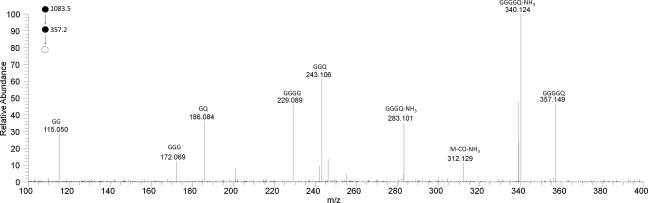
HRMS^3^ spectrum of the *m*/*z* 357 product ion of loss of three AMHO moieties from the protonated octapeptide **1** ion cyclo-[GGGGQ(AMHO)_3_H]^+^. Annotated ions are acylium species.

A product ion was observed from neutral loss of all three AMHO moieties (*m*/*z* 357.1522) consistent with a molecular formula of C_13_H_21_N_6_O_6_^+^ (calcd.: 357.1517), presumably a peptide ion incorporating the remaining amino acid components. This was identified as a GGGGQ acylium species as in the HRMS^3^ spectrum of this ion (Fig. 3) the product ions observed corresponded to acylium ions of peptides comprising combinations of one to three glycines with glutamine, or two to four glycines.^2^

These findings indicated that **1** was novel among siderophores of the ferrichrome class (Renshaw et al., 2002), being an octapeptide rather than a hexapeptide, and incorporating a glutamine moiety. The presence of a glutamine residue in **1** was confirmed by deuterium labeling and low resolution MS. On infusion of a siderophore preparation in D_2_O into the positive electrospray source of the LTQ mass spectrometer, an ion corresponding to d_14_-**1-Fe** (1150.5 *m*/*z*) was observed, consistent with the presence of 13 exchangeable protons in **1-Fe**.

The analysis of these MS*^n^* spectra left unresolved whether the siderophore was of the ferrichrome class or had an ester-linked structure as suggested by the major neutral loss of hydroxyornithine on MS*^n^* (Lee et al., 2010) (above). However, **1** was found to be resistant to mild base hydrolysis conditions reported to cleave the ester-linked siderophore fusigen to its component *cis*-AMHO moieties (Diekmann and Zahner, 1967, Lee et al., 2010). In addition, evidence for a cyclic octapeptide structure with three pendant anhydromevalonyl moieties and thus six free hydroxyl groups was provided by the observation of a hexa-acetate product (*m*/*z* 1335.4) on acetylation of **1** following the precedent of Sharman et al. (1995), and of abundant neutral losses of 30, 60 and 90 u in the MS^2^ of **1-Fe** in negative electrospray ionization mode, consistent with neutral losses of CH_2_O by retro-Aldol fragmentation of each of the three pendant anhydromevalonyl moieties (Simionato et al., 2006).

These further data strongly supported the ferrichrome structure with pendant anhydromevalonyl moieties. However other structural questions remained unresolved. The direction of the amino acid sequence remained to be determined, as did the stereochemistry of the pendant AMHO moieties, indicated to be *trans* by the accumulation of *trans-*rather than *cis*-AMHO in cultures of Δ*sidN* mutant strains (unpublished data, above). These questions have been resolved by NMR analysis of isolated **1**, supported by further interpretation of the mass spectrometric data based on comparative investigations of two related siderophores of known structure, ferrirubin (**4-Fe**) and ferrirhodin (**5-Fe**).

For NMR analysis of **1**, a purified fraction was prepared from a culture of *E. festucae* grown under iron-depleted conditions. Addition of ferric chloride to the culture supernatant to facilitate detection and ion exchange and size-exclusion chromatography afforded an enriched fraction of **1-Fe**. Deferration with 8-hydroxyquinoline converted **1-Fe** to **1**, which was separated by reverse-phase chromatography and analyzed by ^1^H and ^13^C NMR (Supplementary data, Figs. S1–S6).

The assignment of the NMR spectra (Table 1) was carried out with reference to previously published chemical shift data for ferrichrome siderophores (Jalal and van der Helm, 1991). The chemical shifts of the glycine and AMHO moieties were consistent with those reported for other ferrichrome siderophores, and the remaining resonances are consistent with the presence of a glutamine residue. Patterns for resolved multiplets in the ^1^H NMR were consistent with these conclusions. Within the three AMHO moieties, the resonances of equivalent side-chain nuclei were generally unresolved, as were resonances for the three peptide carbonyl nuclei (C18, C21 and C24) and the alpha proton and carbon nuclei (C20, C23) of two of the AMHO residues. The chemical shifts of the 8″ and 9″ (and equivalent) protons and carbons indicated that the configuration of the anhydromevalonyl group in the AMHO residues was *trans* (Jalal and van der Helm, 1991).

**Table 1:** ^1^H and ^13^C NMR data for epichloenin A in DMSO-*d*_6_.

Position	*δ*^13^C (ppm), multi.	*δ*^1^H (ppm)	*J* (Hz)	COSY	NOESY^A^	HMBC
1		8.07	*brt* (5.3)	2a/b	4, 1″″a/b, 2″″a/b, 22, 23	2, 24
2	42.6, CH2	a 3.57	*d* (5.3)	1	4	3, 24
		b 3.80	–B	1	4	3, 24
3	169.4^a^, C					
4		7.83	*o*^C^	5a/b	1, 2a/b, 7	3, 5
5	42.1, CH2	a 3.73	–B	4	7	6
		b 3.81	–B	4	7	6
6	169.7,C					
7		8.29	*brs*	8a/b	4, 5a/b, 10	6, 8
8	42.8, CH2	a 3.68	–B	7	10	9
		b 3.72	–B	7	10	9
9	169.4^a^, C					
10		8.00	*brt* (5.1)	11a/b	7, 8a/b, 13	9, 11
11	42.2, CH2	a 3.76	–B	10	13	12
		b 3.79	–B	10	13	12
12	169.1, C					
13		7.84	*o*^C^	14	10, 11a/b, 16, 1′a/b, 2′	12
14	52.9, CH	4.16	*brdt* (6.0, 7.6)	13, 1′a/b	16, 2′, 1″a/b	15, 1′, 2′
15	172.0, C					
1′	26.8, CH2	a 1.81	*m*	14, 2′	13, 16, 1″a/b	14, 15, 2′, 3′
		b 1.99	*m*	14, 2′	13, 16, 1″a/b	14, 15, 2′, 3′
2′	31.4, CH2	2.13	*t* (8.4)	1′a/b	13, 14, 16, 1″a/b	14, 1′, 3′
3′	173.9, C					
4′		a 6.84	*brs*			2′, 3′
		b 7.32	*brs*			3′
16		8.22	*brs*	17	13, 14, 19, 1′a/b, 2′, 1″a/b, 2″a/b, 3″	
17	53.78, CH	4.02	*brddd* (5.5, 6.5, 8.3)	16, 1″a/b	19, 2″a/b, 3″	18, 1″
18, 21, 24^b^	172.2, C					
19		7.87	*brs*	20	16, 17, 22, 1″a/b, 1‴a/b, 2″a/b, 2‴a/b, 3″, 3‴	
20, 23^c^	53.5, CH	4.07	*brdt* (6.6, 8.5)	19, 22, 1‴a/b, 1″″a/b	1, 2‴a/b, 2″″a/b, 3‴, 3″″	21, 24, 1‴, 1″″, 2″, 2‴, 2″″
22		7.93	*brs*	23	1, 19, 1‴a/b, 1″″a/b, 2‴a/b, 2″″a/b, 3‴, 3″″	
1″	27.8^d^, CH2	a 1.57^e^	*o*^C^	17, 2″a/b	14, 16, 19, 1′a/b, 2′, 3″	17, 18, 2″, 2‴, 2″″, 3″, 3‴, 3″″
		b 1.74	*m*	17, 2″a/b	14, 16, 19, 1′a/b, 2′, 3″	2″, 2‴, 2″″
1‴	27.9, CH2	a 1.57^e^	*o*^C^	20, 2‴a/b	19, 20, 3‴	20, 21, 2″, 2‴, 2″″, 3″, 3‴, 3″″
		b 1.68	*m*	20, 2‴a/b	19, 20, 3‴	2″, 2‴, 2″″
1″″	27.8^d^, CH2	a 1.57^e^	*o*^C^	23, 2″″a/b	1, 22, 3″″	23, 24, 2″, 2‴, 2″″, 3″, 3‴, 3″″
		b 1.73	*m*	23, 2″″a/b	1, 22, 3″″	2″, 2‴, 2″″
2″, 2‴, 2″″^f^	23.2, CH2	a 1.50	*m*	1″a/b, 1‴a/b, 1″″a/b, 3″, 3‴, 3″″	1, 16, 17, 19, 20, 22, 23	1″, 1‴, 1″″, 3″, 3‴, 3″″
		b 1.57^e^	*m*	1″a/b, 1‴a/b, 1″″a/b, 3″, 3‴, 3″″	1, 16, 17, 19, 20, 22, 23	1″, 1‴, 1″″, 3″, 3‴, 3″″
3″, 3‴, 3″″^g^	46.5, CH2	3.49	*m*	2″a/b, 2‴a/b, 2″″a/b	16, 17, 19, 20, 22, 23, 1″a/b, 1‴a/b, 1″″a/b, 6″, 6‴, 6″″	1″, 1‴, 1″″, 2″, 2‴, 2″″, 5″, 5‴, 5″″
4″, 4‴, 4″″^h^		9.64	*brs*			
5″, 5‴, 5″″^i^	166.5, C					
6″, 6‴, 6″″^j^	116.1, CH	6.22	*s*	8″, 8‴, 8″″, 9″, 9‴, 9″″	3″, 3‴, 3″″	
7″, 7‴, 7″″^k^	151.1, C					
8″, 8‴, 8″″^l^	18.2, CH3	2.02	*s*	6″, 6‴, 6″″	9″, 9‴, 9″″, 10″, 10‴, 10″″	6″, 6‴, 6″″, 7″, 7‴, 7″″, 9″, 9‴, 9″″
9″, 9‴, 9″″^m^	43.8, CH2	2.23	*t* (6.5)	6″, 6‴, 6″″, 10″, 10‴, 10″″	8″ 8‴, 8″″	6″, 6‴, 6″″, 7″, 7‴, 7″″, 8″, 8‴, 8″″, 10″, 10‴, 10″″
10″, 10‴, 10″″^n^	59.1, CH2	3.54	*t* (6.5)	9″, 9‴, 9″″	8″, 8‴, 8″″	7″, 7‴, 7″″, 9″, 9‴, 9″″

A Strong NOE peaks listed. Cross-peaks also observed in the COSY spectra are not listed.

B Signals obscured by residual H_2_O (3.75 ppm) assigned from COSY.

C Overlapping peaks assigned from COSY.

a–n Resonances indistinguishable in spectra.

Correlations in the 2D COSY, NOESY, edited HSQC and HMBC spectra were consistent with a structure of the ferrichrome class for **1** (Table 1). The tetraglycylglutamine sequence inferred from the MS data is supported by the COSY and ^2^J HMBC connectivity, although some signals are coincident or near-overlapping. N-acylation of the terminal glycine α-amino group by an AMHO carboxyl was established by HMBCs between AMHO C=O (unresolved signals, *δ* 172.2 ppm), and N1–H (^2^J coupling) and C2–H_a_ and C2–H_b_ (^3^J coupling) of the terminal glycine. While ^3^J heteronuclear couplings were also observed within side-chain residues, these were the only such couplings observed for ring residues, presumably due to conformational constraints.

While the set of linkages established by observed HMBCs between adjacent residues was incomplete, NOEs were observed between each of the amide protons and protons of the corresponding i-1 residue around the ring (Table 1). Additional NOEs involving side-chain protons were also observed confirming the acyl linkage between AMHO and the N-terminal glycine, and N-acylation of the N-terminal AMHO α-amino group by the glutamine carboxyl. In the latter case NOE correlations were observed between the glutamine methine (C14-H) and C1′- and C2′-methylene protons and the C1″-methylene protons of the adjacent AMHO side-chain, confirming the proximity of these moieties. Thus the combination of HMBC and NOESY data confirmed the peptide composition and sequence as determined by MS*^n^* (above) and established the direction of the peptide sequence is as shown in **1**.

**Fig. 4:**
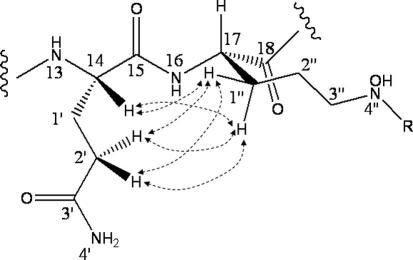
Schematic model showing the proposed peptide ring conformation of **1** in the vicinity of the C14-glutamine and adjacent C17-AMHO moieties, and key NOESY correlations within one of the accessible side-chain rotamers.

The NOESY data also provided evidence of the relative configurations of the glutamine and AMHO moieties. Whereas NOEs were observed between C14–H on the glutamine α-carbon, and the proximal AMHO side-chain methylene protons (C1″–H_a_ and H_b_) (above), no NOE was observed between C14–H and the adjacent AMHO Cα-proton C17–H, nor between C17–H and the glutamine side-chain methylene protons. Examination of molecular models suggests **1** to be highly conformationally mobile and we have not attempted to define a fully optimized structure. However, models indicate that these NOESY observations can be accounted for if the configurations at C14 and C17 are the same so that the glutamyl and AMHO side-chains are both *cis* with respect to the peptide ring, and **1** can adopt a conformation (Fig. 4) in which C14–H is pseudo-equatorial and proximal to the side-chain of the adjacent AMHO while C17–H is pseudo-axial and remote from the glutamine side-chain.

NOEs were observed between protons along the AMHO side-chain, including between the olefinic proton, C6‴-H, and both the C8‴ methyl protons and C9‴ methylene protons. However closer examination of the data revealed the NOE between C6‴-H and the C9‴ methylene protons was much the greater of these (Supplementary data, Fig. SI 6a), consistent with the *trans* configuration of the anhydromevalonyl moieties deduced from the chemical shift data.

Evidence for the absolute stereochemistry was provided by the CD spectrum of **1-Fe** (Supplementary data, Fig. SI 7), in which a positive band was observed at 465 nm similar to that reported for ferrichrome (van der Helm and Winkelmann, 1994). These data are consistent with **1** having the peptide structure GGGGQ [*trans*-AMHO]_3_ linked as a cycle between *trans*-AMHO and the terminal glycine, with the chiral amino acids having the L (S) configuration (**1**) as for other reported naturally-occurring siderophores of the ferrichrome class (Budzikiewicz, 2010).

The octapeptide **1** is novel among ferrichrome siderophores in incorporating eight amino acids in the peptide ring, and in both the presence of a glutamine residue, and its location upstream of the three acylhydroxyornithine moieties, a position occupied by a conserved glycine in all previously reported ferrichrome siderophores (Budzikiewicz, 2010). The occurrence of a glycine at this position has been related to the conformational constraints of the hexapeptide ring in ferrichrome siderophores (van der Helm et al., 1987), and these may be relaxed for the octapeptide ring in **1-Fe**. These findings exemplify that while biosynthetic gene sequence evidence may provide a lead to a product structure class, as here to a siderophore of the ferrichrome class, inference from gene homologies cannot predict the structure of a product with novel features.

It remained to reconcile the analysis of the MS and NMR data for **1** to establish their consistency and in order to apply MS methodology to elucidate the structure of the related compound **2** (below), not available on a scale sufficient for NMR analysis. This has been achieved by investigation of the HRMS*^n^* of the desferri-forms **4** and **5** (Fig. 1) of the related isomeric ferrichrome siderophores, ferrirubin (**4-Fe**), with pendant *trans-*AMHO moieties, the structure of which has been established unambiguously by X-ray crystallography (Barnes et al., 1985), and ferrirhodin (**5-Fe**) with pendant *cis-*AMHO moieties (Renshaw et al., 2002). Published mass spectrometric data for **4-Fe** and **5-Fe** (Mawji et al., 2008) did not reveal distinctive features associated with the differing configuration of the AMHO moieties. However, we have now acquired HRMS*^n^* data for desferrirubin (**4**) and desferrirhodin (**5**) and found them to show distinctively different patterns of fragmentation.

On HRMS^2^ under positive electrospray conditions **4** undergoes fragmentation (Fig. 5a) to a sequence of product ions analogous to those seen for **1**. A distinctive neutral loss of an inner hydroxyornithine with retention of all three anhydromevalonyl moieties was observed as for **1**. In addition, distinctive product ions were observed corresponding to neutral losses of *trans-*anhydromevalonyl and a serine residue and a *trans*-AMHO serine dipeptide moiety (Fig. 5a, inset). These products of selective cleavage of the peptide ring at the amino acid residue N-acylated by AMHO are equivalent to the neutral losses including a single glycine observed for **1** (Fig. 2). By contrast for **5** with pendant *cis-*AMHO groups, the HRMS^2^ spectrum (Fig. 5b) is relatively sparse, with neutral losses of one to three anhydromevalonyl moieties evident together with a minor neutral loss of anhydromevalonyl plus water, but little evidence of independent loss of hydroxyornithine, nor of selective neutral loss of either serine or glycine with *cis-*anhydromevalonyl or *cis-*AMHO. While the reaction mechanisms facilitating the neutral loss of an inner ornithine and selective losses involving the N-terminal residue of the proteinogenic amino acid residues in the HRMS^2^ of **1** and **4** remain to be elucidated, the occurrence of these processes appears to be a useful characterizing feature of the MS*^n^* of desferrichrome siderophores with pendant *trans*-AMHO groups. In particular, the observation of these fragmentation patterns confirms the consistency of the MS and NMR data for **1** both in terms of the stereochemistry of the pendant AMHO moieties and the direction of the peptide sequence.

**Fig. 5a:**
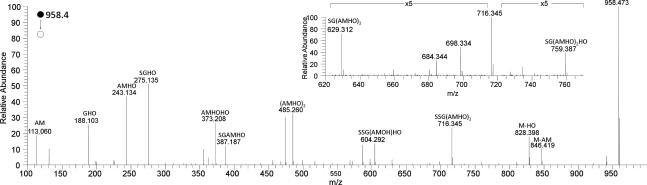
HRMS^2^ spectrum of **4**. Annotated ions are acylium species. Inset is subsection of the spectrum, showing the losses of serine corresponding to glycine losses in **1**.

**Fig. 5b:**
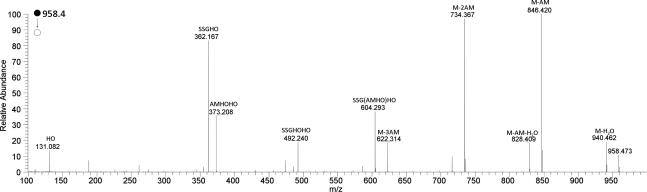
HRMS^2^ spectrum of **5**. Annotated ions are acylium species.

The confirmation that the configuration of the AMHO moieties in **1** is *trans*-rather than *cis-*raises questions about the specificity of the third adenylation domain (SidNA3) of the SidN NRPS. The synthetic protein was shown to activate *cis-*AMHO but not ornithine or any of the 20 proteinogenic amino acids (Lee et al., 2010). However *trans*-AMHO was not tested in these experiments because of difficulties sourcing this unusual amino acid. The recently determined structure of SidNA3 showed that it has a very large substrate binding pocket that may be able to accommodate both isomers with similar specificity (Lee et al., 2010). Efforts are currently underway to test the relative specificity of SidNA3 towards the *cis-* and *trans*-isomers of AMHO. The selective formation by *E. festucae* of **1** rather than its *cis*-AMHO isomer may be the consequence of selective biosynthesis of *trans-*AMHO by this fungus, as demonstrated in the Δ*sidN* mutant (unpublished data), rather than of the specificity of the enzyme.

### 2.2 Epichloënin B

With the elucidation of the structure of **1**, we were able to deduce the structure of a related siderophore we have observed by LC-MS but have not isolated in sufficient quantity for NMR analysis. In low resolution LC-MS*^n^* analysis of supernatants of WT *E. festucae* Fl1 cultures grown under iron-depleted conditions a minor component was noted as co-eluting ions in the MS^1^ trace of *m*/*z* 514 ([M+2H]^2+^) and *m*/*z* 1026.5 ([M+H]^+^), eluting slightly later than **1** and corresponding to a variant with three rather than four glycines. We have designated this resolved compound as epichloënin B and shown it to have the structure **2** (Fig. 1). Evidence that **2** like **1** was a product of the NRPS SidN was provided by LC-MS examination of cultures of Δ*sidN* and complemented (C*-sidN*) mutant strains of *E. festucae* (unpublished data) which showed that **2** was present in cultures of the complemented strain as for the WT, but not in cultures of the deletion mutant strain. The molecular formula of **2** was determined to be C_44_H_71_N_11_O_17_ by positive electrospray HRMS*^n^* (*m*/*z* 1026.5092 [M+H]^+^). An ion corresponding to the putative Fe-bound form (**2-Fe**) co-eluted with **1-Fe**, but MS and MS*^n^* data for this species was ambiguous as it could not be resolved from a source fragment of identical *m*/*z* arising from neutral loss of glycine from **1-Fe**.

**Fig. 6:**
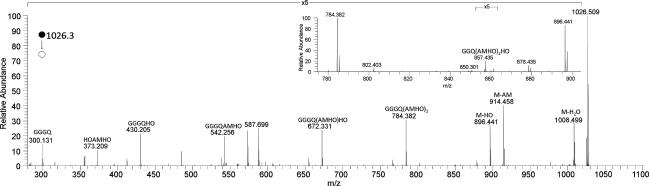
HRMS^2^ spectrum of the protonated heptapeptide **2** ion cyclo-[GGGQ(AMHO)_3_H]^+^, using Collision Induced Dissociation with different amount of energy. Annotated ions are acylium species. Inset is a subsection of the spectrum, showing the loss of AM plus glycine.

The HRMS*^n^* spectrum of **2** (*m*/*z* 1026.5) (Fig. 6) showed a similar series of major product ions corresponding to neutral losses of water, anhydromevalonyl, hydroxyornithine and AMHO residues and combinations of these as described for **1** above. As for **1**, a product ion from the selective neutral loss of glycine and anhydromevalonyl was observed (inset, Fig. 6). Similarly, a product ion from the loss of three AMHO moieties was also observed, in this case corresponding to the acylium ion of the tetrapeptide GGGQ (Fig. 6). HRMS^3^ of this ion (*m*/*z* 300) afforded as a single major product a glycylglutamine dipeptide acylium ion (*m*/*z* 186.0867 [QG]^+^ (calcd. for C_7_H_15_N_3_O_3_^+^, 186.0873)) as observed in the HRMS^2^ and HRMS^3^ spectra of **1**. These MS*^n^* data are consistent with **2** being a heptapeptide variant of **1** with three rather than four glycines in the ring. In particular the observation in the MS^2^ spectrum of a distinctive neutral loss of hydroxyornithine, as seen for **1** and **4**, is consistent with **2** being a *trans-*AMHO ferrichrome siderophore, and the observation of selective neutral losses of glycine together with anhydromevalonyl, as seen for **1**, is consistent with acylation of the N-terminal glycine of the proteinogenic amino acid sequence by AMHO.

The biosynthesis by *E. festucae* of the minor siderophore **2** together with **1** is probably of minor significance for siderophore functionality in the endophyte grass symbiosis, but is of interest in demonstrating a degree of non-specificity of the iterative condensation process of the SidN enzyme complex. Further indications of a degree of non-specificity have been provided by the detection at very low levels in LC-MS*^n^* analyses of culture medium extracts of minor variants apparently incorporating two glutamine residues with varying numbers of glycine residues (unpublished data).

Compound **2** is also unusual among ferrichrome siderophores both as a heptapeptide, and as with **1** in the presence and location of the glutamine residue (Renshaw et al., 2002) and as for **1** (above) this may relate to relaxed conformational constraints in the peptide ring. These unusual features of these *Epichloë* siderophores raise interesting questions about their phylogeny and functionality, as speciation of siderophores has been attributed to microbial competition (Renshaw et al., 2002) which appears unlikely to be a major factor in the protected environment of the plant apoplast.

### 2.3 Iron-bound epichloënin A and epichloëamide *in planta*

**Fig. 7:**
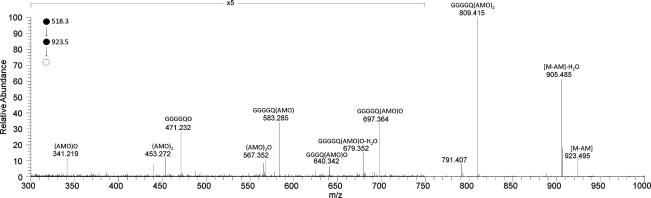
HRMS^3^ spectrum of the *m*/*z* 923 product ion of loss of an anhydromevalonyl moiety from the protonated octapeptide **3** ion cyclo-[GGGGQ(AMO)_3_H]^+^. Annotated ions are acylium species.

In prior investigations of endophyte metabolites we did not detect **1** or **1-Fe** in metabolomic comparisons of extracts of leaf, pseudostem and seeds of endophyte-infected and endophyte-free perennial ryegrass using direct infusion MS (DIMS) (Cao et al., 2008, Koulman et al., 2007b), nor in a DIMS comparison of extracts of leaf and pseudostem of perennial ryegrass infected with WT or Δ*sidN* mutant strains (unpublished data). However, we were able to detect epichloënin A (**1-Fe**) in guttation fluid from ryegrass (*L. perenne*) plants infected with WT *E. festucae*, but not in plants infected with a Δ*sidN* mutant strain, and to show *in planta* production of **1** was restored by complementation of Δ*sidN* (C-*sidN*) (unpublished data). In the course of this study we also detected another peptide, eluting earlier in LC-MS, which showed the same pattern of occurrence, being present in guttation fluid from plants infected with the WT and C-*sidN* strains, but not Δ*sidN* mutant strains and exhibiting a related but differing pattern of fragmentation on MS*^n^*. This was detected by HRMS as the [M+2H]^2+^ ion (*m*/*z* 518.2792), consistent with the formulation of the peptide as epichloëamide **3** (C_46_H_74_N_12_O_15_), a reduction product of **1**. The HRMS^2^ spectrum (Section 3) showed a major high mass product ion from the neutral loss of anhydromevalonyl, but a product ion from the neutral loss of an ornithine residue was present at only low abundance in contrast to the facile loss of hydroxyornithine from the parent ion in the case of **1** and **2** (and **4**). The HRMS^3^ spectrum of the product ion of neutral loss of anhydromevalonyl from **3** (*m*/*z* 923) (Fig. 7 and Section 3) revealed a series of major product ions from the neutral loss of loss of water, anhydromevalonyl, and anhydromevalonylornithine (AMO) moieties and combinations of these species, together with the complementary product ions. A product ion corresponding to the acylium ion of a hexapeptide ([GGGGQO]^+^) incorporating the four proteinogenic amino acid residues and an ornithine was observed but lower mass fragments deriving from further cleavage of this peptide were not observed. However products of cleavage near both ends of the sequence were detected in the HRMS^3^ spectrum of **3**, corresponding to losses of an AMO glycine dipeptide residue ([GGGQ(AMO)O]^+^), and a glycylglutamylornithine tripeptide residue ([GGG(AMO)_2_]^+^), respectively. Thus we did not observe for **3** the structurally distinctive features of the HRMS*^n^* of **1**, **2** and **4**, namely the selective cleavage of the N-terminal proteinogenic amino acid acylated by *trans*-AMHO, nor the major neutral loss of an inner amino acid (in this case ornithine) to form a product ion retaining all three *trans*-anhydromevalonyl moieties. Evidently these processes are dependent on the *trans*-anhydromevalonyl hydroxamate structures in the siderophores rather than the presence of pendant *trans*-anhydromevalonyl groups alone. Consequently these HRMS*^n^* data for **3** do not provide independent evidence of the peptide sequence or the configuration of the AMHO moieties. However they are consistent with structure **3**, and the assignment of this structure then rests on the structure of **1** as determined above, and the relationship between **1-Fe** and **3** established by their pattern of co-occurrence in plants infected with WT and mutant strains.

We did not detect **3** in initial untargeted LC-MS investigations of *E. festucae* cultures. However subsequent to its discovery in guttation fluid, we were able to detect **3** at low concentrations by LC-MS^3^ in both culture supernatant and mycelial extracts from WT *E. festucae* cultures but not from the Δ*sidN* mutant. The identity of **3** from guttation fluid and fungal cultures was confirmed by co-elution and MS^3^ spectrum similarity. The biosynthetic origin of **3** remains to be elucidated and is the subject of ongoing research. From its structure and pattern of co-occurrence with **1-Fe** we infer **3** is a siderophore reduction product, and as such it may be a by-product of reductive uptake by the fungus of Fe from **1-Fe** (van der Helm and Winkelmann, 1994).

### 2.4 Conclusions

We have established that the endophytic fungus *E. festucae* synthesizes extracellular siderophores **1** and **2** of unusual structure compared to other ferrichrome siderophores. This raises a number of interesting questions about the biosynthesis of the siderophore by the SidN enzyme complex of this fungus, and the biological significance of these distinctive structures in view of the symbiotic relationship of *E. festucae* with its plant host. With the detection of **1-Fe** and its putative metabolic by-product **3** in guttation fluid of *E. festucae*-infected plants we have confirmed that extracellular siderophore biosynthesis is active within the symbiotum. The functional role of **3** in the symbiotum, if any, remains unclear and its occurrence in the guttation fluid of endophyte-infected plants remains to be accounted for. However the Fe retrieval function of **1-Fe** is apparent and its significance to the symbiosis has been established in studies with the Δ*sidN* mutant strain reported elsewhere (Johnson et al., 2007a; unpublished data). The observation of **1-Fe** in guttation fluid is of considerable interest, as it demonstrates mobility of the siderophore within the plant, incomplete recovery of siderophore-chelated iron by the fungus, and the loss of iron by the symbiosis as a whole. We have not attempted a quantitative estimate but the levels appeared to be extremely low. However circulating levels of chelated iron (e.g. as a citrate complex (Rellan-Alvarez et al., 2010)) in plants are evidently normally very low, and the implications for the plant of a competing fungal iron-chelating agent mobile in the plant requires further investigation. While associations of *Epichloë* and related *Neotyphodium* fungal endophytes with their grass hosts are characterized as asymptomatic (Clay and Schardl, 2002), a weak resistance response has been suggested to occur in endophyte-infected grass plants (Rasmussen et al., 2008) and siderophore elicitor activity (Dellagi et al., 2009) may play a role in this.

## 3 Experimental

### 3.1 General procedures

Preparative HPLC was performed on a System Gold instrument (Beckman Coulter) with a UV absorbance detector. Low resolution electrospray ionization (ESI) mass spectral data were acquired on a Thermo LTQ linear ion trap mass spectrometer either by direct infusion or by HPLC using a Thermo Surveyor LC system equipped with an in-line photodiode array (PDA) detector for ultraviolet–visible (UV–visible) detection. HRMS*^n^* spectra were obtained with a Thermo LTQ Orbitrap Velos with chip based nanospray infusion (Advion Triversa Nanomate). NMR spectra were recorded on a Bruker AV600 NMR spectrometer (600 and 150 MHz for ^1^H and ^13^C NMR, respectively) equipped with a triple resonance TXI room temperature probe in DMSO-*d*_6_ (Sigma), and calibrated to the residual solvent peaks (*δ*^13^C 39.5 ppm, *δ*^1^H 2.50 ppm). Circular dichroism spectral data were acquired on an Applied Photophysics PiStar-180 spectrometer. Molecular modeling was carried out with Chem3D software (Perkin–Elmer Informatics, Cambridge Massachusetts, USA).

### 3.2 Chemicals

Desferrirubin (**4**) and desferrirhodin (**5**) were obtained from Genaxxon bioscience (Ulm, Germany). Milli-Q water and HPLC grade MeOH and MeCN (Merck) were used for HPLC. Open column chromatography was carried out on Amberlite XAD16 resin (Sigma).

### 3.3 Biological materials

The endophytic fungus *E. festucae* strain Fl1 (WT) used in this study was originally isolated from *Festuca trachyphylla* (*Festuca longifolia*) cv SR-3000 (Siegel et al., 1990) and subsequently maintained in plants of *L. perenne* cv “Grasslands Nui” (seed stored in the Margot Forde Forage Germplasm Centre, AgResearch Grasslands, Palmerston North, New Zealand, Accession No. A 17331) from which it was re-isolated for further investigation. For the characterization of the extracellular siderophore, a 150 ml liquid culture of *E. festucae* strain Fl1 (WT) was grown under iron-depleted conditions for approximately 4 weeks at 22 °C in modified Mantle media (Mantle and Nisbet, 1976), with yeast extract replaced with 0.6 μM thymine and 0.1 mM bathophenanthrolinedisulfonic acid added. Details of DNA isolation from the WT strain, the sequencing and analysis of the *sidN* gene, and of the construction of Δ*sidN* deletion and C-*sidN* complementation mutant constructs, and their inoculation into perennial ryegrass are reported elsewhere (Johnson et al., 2007a; unpublished data). For *in planta* investigations, guttation fluid was collected as previously described (Koulman et al., 2007a) from associations of perennial ryegrass (*L. perenne*) cultivars “G1057” or “Nui” infected with WT, Δ*sidN* or C-*sidN* strains. In brief, plants were placed overnight in a closed container and in the early morning the fluid accumulated at the leaf tips of a plant was collected with a glass pipette, transferred to a plastic container, and stored at −20 °C until analysis.

### 3.4 Siderophore analysis by LC-PDA-MS*^n^*

Samples of supernatant from liquid cultures were separated by centrifugation, and enriched fractions prepared by solid-phase extraction following the procedure of McCormack et al. (2003). All samples were stored at −20 °C prior to analysis. The samples were thawed prior to analysis and transferred to a HPLC vial with 200 μl insert. Samples were kept at 4 °C in the autosampler, and 20 μl subsamples were injected. Analytes were eluted through a C18 Luna column (Phenomenex Torrence, CA, USA) (150 × 2 mm, 5 μm) at a flow rate of 200 μl min^−1^ with a solvent gradient (gradient 1) (solvent A: H_2_O 0.1% formic acid; B: MeCN 0.1% formic acid), starting with 5% B, 95% A for 5 min and then increasing to 33% B after 15 min, then to 95% B by 20 min where it was held for 5 min to wash the column before being returned to 5% B and allowed to re-equilibrate. Mass spectra were detected using ESI in positive ion mode. The spray voltage was 4.5 kV and the capillary temperature 275 °C. The flow rates of nitrogen sheath gas, auxiliary gas, and sweep gas were set to 20, 5, and 10 (arbitrary units), respectively. Epichloënin A (**1**) was detected by UV absorbance at 245 nm and co-eluting MS^1^ ions of *m*/*z* 542.5 ± 0.5 [M+2H]^2+^ and *m*/*z* 1083 ± 0.5 [M+H]^+^; ferriepichloënin A (**1-Fe**) was detected by UV absorbance at 440 nm and co-eluting MS^1^ ions of *m*/*z* 568.5 ± 0.5 [M+2H]^2+^ and *m*/*z* 1136 ± 0.5 [M+H]^+^; epichloënin B (**2**) was detected as co-eluting MS^1^ ions of *m*/*z* 514 ± 0.5 [M+2H]^2+^ and *m*/*z* 1026.5 ± 0.5 [M+H]^+^.

For targeted analysis of guttation fluid in selective reaction monitoring mode, epichloëamide (**3**) was detected by selecting and fragmenting the parent ion *m*/*z* 518.5 ± 2 [M+2H]^2+^ (35% relative collision energy), and selecting and fragmenting the product ion *m*/*z* 923.3 ± 2 (35% relative collision energy) and monitoring the total ion current: ferriepichloënin A (**1-Fe**) was detected by selecting and fragmenting the parent ion *m*/*z* 568.5 ± 2 [M+2H]^2+^ (35% relative collision energy), and selecting and fragmenting the product ion *m*/*z* 1024 ± 2 (35% relative collision energy) and monitoring the total ion current.

Low resolution negative ion MS*^n^* data for **1-Fe** was acquired by direct infusion of an enriched sample into the mass spectrometer with setting as above except for a negative spray voltage of 4.5 kV, and selecting and fragmenting the parent ion *m*/*z* 1134.5 ± 2 [M−H]^−^ (35% relative collision energy).

The acetylation products of **1** (below) were analyzed by LC-MS with the same solvents and other instrument settings as above except with a modified gradient (gradient 2): starting with 5% B, 95% A for 2 min and then increasing to 95% B by 25 min where it was held for 5 min to wash the column before being returned to 5% B and allowed to re-equilibrate. Epichloënin A hexa-acetate (**1**-hexa-acetate) was detected in the MS^1^ trace at *m*/*z* 1335.5 [M+H]^+^.

### 3.5 HRMS*^n^*

High resolution mass spectra were obtained through nanospray ionization. Samples of enriched fractions prepared by solid-phase extraction of culture supernatants (above) were analyzed with the LTQ-Orbitrap Velos (Thermo Scientific, Hemel Hampstead, UK) coupled to the Triversa Nanomate system Advion BioSciences, Inc., Ithaca, NY, USA). The Triversa Nanomate infused 5 μl samples with gas pressure of 0.2 psi and a voltage of 1.2 kV. The LTQ-Orbitrap Velos was controlled manually and scan events were decided on the fly. Generally parent ions of selected masses were isolated with a 1.5 *m*/*z* width in the dual pressure linear ion trap and then fragmented either in the linear ion trap with 35% relative collision energy to acquire MS^2^ data for higher mass fragments and MS^3^ data, or in the HCD collision cell, using a range of collision energies of from 5% to 75% relative collision energy to provide coverage in the MS^2^ spectrum of the low *m*/*z* range below the low mass cut-off (1/3 of parent *m*/*z*) of the linear ion trap. Average MS^2^ spectra from these combined data are reported in Figs. 2, 5a, b and 6. CID spectra obtained with a single energy setting in the linear ion trap are reported below. All spectra were recorded in the Orbitrap set at 100,000 resolution. Due to the very low concentration of the siderophores the calibration was sub-optimal leading to poor mass accuracy: deviations between calculated and observed *m*/*z* ratios were generally within 5 ppm for MS^2^ spectra, but of the order of 10 ppm for MS^3^ spectra. However the accuracy of the *m*/*z* differences between spectral features was less affected (deviations generally within 2–3 ppm) and these data were therefore used to support assignment of molecular formulae to product ions and neutral losses.

### 3.6 Isolation of epichloënin A (**1**)

The culture supernatant was separated by centrifugation and 5 mM FeCl_3_ was added to visualize the siderophore. The bulk of the supernatant (120 ml) was loaded on a solid-phase extraction cartridge containing Amberlite XAD16 resin. The resin was washed with water and the dark orange siderophore eluted with methanol. The fraction was evaporated to dryness (50 mg), redissolved in water and purified by size exclusion chromatography on a Bio-gel P-2 (Bio-Rad) column (1.6 × 60 cm). Deferration of the siderophore was accomplished by treatment with 8-hydroxyquinoline and heating to 60 °C for 30 min. The 8-hydroxyquinoline was removed by chloroform extraction (5×) and the aqueous phase was further purified by HPLC on a C18 Luna column (Phenomenex) (150 × 3 mm, 3 μm) at a flow rate of 0.3 ml min^−1^ with a solvent gradient (solvent A: H_2_O 0.1% TFA; B: MeCN 0.1% TFA), starting with 0% B, 100% A for 2 min and then increasing to 12% B by 3 min, then to 16% B by 19 min and to 95% B by 20 min where it was held for 6 min to wash the column before being returned to 0% B and allowed to re-equilibrate. Detection was by monitoring absorbance at 220 nm. The siderophore fraction (Rt 16.5 min) was dried using a Savant SPD112 concentrator (Thermo Scientific) to remove the acetonitrile to give a colorless gum (0.9 mg, estimated by NMR) which was re-dissolved in DMSO-*d*_6_ for NMR analysis. 1D ^1^H and ^13^C spectra and 2D COSY, NOESY, edited HSQC and HMBC spectra were recorded.

### 3.7 Base treatment and acetylation of **1**

An enriched aqueous fraction of **1** was adjusted to pH 12.0 with 1 N NaOH and the mixture incubated at room temperature for 1 h and then overnight at 4 °C, conditions for the hydrolysis of fusigen (Diekmann and Zahner, 1967, Lee et al., 2010). The solution was neutralized with 1 N HCl and analysis by LC-MS showed ca. 90% recovery of **1**.

A fraction of **1** was dissolved in pyridine–acetic anhydride and allowed to react at room temperature for 4 h (Sharman et al., 1995). An aliquot was taken up in MeCN:H_2_O (1:1) and the formation of **1**-hexa-acetate was shown by LCMS analysis.

### 3.8 Epichloënin A (**1**)

Colorless gum. UV (MeCN–H_2_O) *λ*_max_ 245 nm: analytical HPLC: Rt = 14.15 min (gradient 1): ^1^H, ^13^C spectra and 2D NMR data, see Table 1: HRMS (positive ESI) *m*/*z*: 1083.5315 [M+H]^+^ (calcd. for C_46_H_75_N_12_O_18_^+^, 1083.5317). HRMS^2^ (CID) 1083.5 @ 35% CE: 1065.5211 [M+H−H_2_O]^+^ (calcd. for C_46_H_73_N_12_O_17_^+^, 1065.5211) (100), 971.4804 [M+H−AM]^+^ (calcd. for C_40_H_67_N_12_O_16_^+^, 971.4793) (38), 953.4571 [M+H−HO]^+^ (calcd. for C_41_H_65_N_10_O_16_^+^, 953.4575) (64), 841.4058 [GGGGQ(AMHO)_2_]^+^ (calcd. for C_35_H_57_O_14_N_10_^+^, 841.4050) (58), 823.3952 [GGGGQ(AMHO)_2_−H_2_O]^+^ (calcd. for C_35_H_55_O_13_N_10_^+^, 823.3950) (10), 729.3532 [GGGGQ(AMHO)HO]^+^ (calcd. for C_29_H_49_N_10_O_12_^+^, 729.3526) (37), 711.3291 [GGGGQ(AMHO)AM]^+^ (calcd. for C_30_H_47_N_8_O_12_^+^, 711.3308) (8), 599.2787 [GGGGQAMHO]^+^ (calcd. for C_24_H_39_N_8_O_10_^+^, 599.2784) (28), 487.2263 [GGGGQHO]^+^ (calcd. for C_18_H_31_N_8_O_8_^+^, 487.2259) (27), 430.2050 [GGGQHO]^+^ (calcd. for C_16_H_28_N_7_O_7_^+^, 430.2045) (10), 357.1522 [GGGGQ]^+^ (calcd. for C_13_H_21_N_6_O_6_^+^, 357.1517) (3), 340.1257 [GGGGQ-NH_3_]^+^ (calcd. for C_13_H_18_N_6_O_5_^+^, 340.1251) (3); for other data see Fig. 2, which is the average of the spectra obtained by HRMS^2^ (CID @ 35% CE, HCD @ 10, 15, 20, 25, 35, 40 and 45 eV) and Fig. 3, average of the spectra obtained by HRMS^3^ (CID) 1083.5 @35% CE; (CID) 357 @ 35% CE) and HRMS^3^ (CID) 1083.5 @35% CE; (HCD) 357 @ 35% CE.

#### 3.8.1 Hexa-acetate

Analytical HPLC: Rt = 14.47 min (gradient 2): MS*^n^* (positive ESI) *m*/*z:* 1335.5 [M+H]^+^, MS^2^ (CID) 1335.5 @ 35% CE: 1317 [M+H−H_2_O]^+^ (100), 1293 [M+H−CH_2_CO]^+^ (89), 1275 [M+H−CH_3_CO_2_H]^+^ (55), 1181 [M+H−AM−CH_2_CO]^+^ (84), 1163 [M+H−HO−CH_2_CO]^+^ (32), 1124 [M+H−G−AM−CH_2_CO]^+^ (10), 1121 [M+H−HO−2CH_2_CO]^+^ (19), 1103 [M+H−HO−CH_2_CO−CH_3_CO_2_H]^+^ (12), 1009 [M+H−AMHO−2CH_2_CO]^+^, (13), 855 [M+H−AMHO−AM−3CH_2_CO]^+^ (11).

### 3.9 Ferriepichloënin A (**1-Fe**)

UV (MeCN–H_2_O) *λ*_max_ 440 nm: CD (H_2_O, *c* 8.0 × 10^−4^ M) 465 (+2.9) nm (Δ*ε*): analytical HPLC: Rt = 15.45 min (gradient 1): HRMS*^n^* (positive ESI) *m*/*z* 1136.4436 [M(^56^Fe)+H]^+^ (calcd. for C_46_H_72_^56^FeN_12_O_18_^+^, 1136.4432) (100), 1134.4450 [M(^54^Fe)+H]^+^ (calcd. for C_46_H_72_^54^FeN_12_O_18_^+^, 1134.4478) (6), HRMS^2^(CID) 1136.5 @ 35% CE: 1118.4276 [M+H−H_2_O]^+^ (calcd. for C_46_H_70_^56^FeN_12_O_17_^+^, 1118.4326) (100), 1108.443 [M+H−CO]^+^ (18), 1005.427 (30), 894.3138 [M+H−AMHO]^+^ (calcd. for C_35_H_54_^56^FeN_10_O_14_^+^, 894.3165) (20), 538.1695 [Fe−(AMHO)_2_]^+^ (calcd. for C_22_H_34_^56^Fe N_4_O_8_^+^, 538.1721) (25). MS*^n^* (negative ESI) *m*/*z:* 1134.4 [M−H]^−^, MS^2^ (CID) 1134.5 @ 35% CE: 1116.4 [M−H−H_2_O]^−^ (24), 1104.5 [M−H−CH_2_O]^−^ (100), 1098.1 [M−H−2H_2_O]^−^ (10), 1086.6 [M−H−CH_2_O−H_2_O]^−^ (29), 1074.2 [M−H−2CH_2_O]^−^ (23), 1044.3 [M−H−3CH_2_O]^−^ (7), 1022.3 [M−H−AM]^−^ (17), 1004.3 [M−H−HO]^−^ (48), 992.3 [M−H−AM−CH_2_O]^−^ (23), 986.6 [M−H−HO−H_2_O]^−^ (18), 974.6 [M−H−HO−CH_2_O]^−^ (25), 956.1 [M−H−HO−H_2_O−CH_2_O]^−^ (18), 842.3 (10), 677.5 (7).

### 3.10 Epichloënin B (**2**)

Analytical HPLC: Rt = 14.27 min (gradient 1): HRMS (positive ESI) *m*/*z*: 1026.5092 [M+H]^+^ (calcd. for C_44_H_72_N_11_O_17_^+^, 1026.5102), HRMS^2^ (CID) 1026.5 @ 35% CE: 1008.5000 [M+H−H_2_O]^+^ (calcd. for C_44_H_70_N_11_O_16_^+^, 1008.4997) (100.00), 914.4577 [M+H−AM]^+^ (calcd. for C_38_H_64_N_11_O_15_^+^, 914.4578) (44), 896.4389 [M+H−HO]^+^ (calcd. for C_39_H_62_N_9_O_15_^+^, 896.4365) (96), 784.3833 [GGGQ(AMHO)_2_]^+^ (calcd. for C_33_H_54_O_13_N_9_^+^, 784.3836) (81), 766.3702 [GGGQ(AMHO)_2_−H_2_O]^+^ (calcd. for C_33_H_52_O_12_N_9_^+^, 766.3730) (15), 672.3310 [GGGQ(AMHO)HO]^+^ (calcd. for C_27_H_46_N_9_O_11_^+^, 672.3311) (46), 654.3107 [GGGQ(AMHO)AM]^+^ (calcd. for C_28_H_44_N_7_O_11_^+^, 654.3093) (14), 573.2190 (8), 542.2558 [GGGQAMHO]^+^ (calcd. for C_22_H_36_N_7_O_9_^+^, 542.2569) (30), 485.2570 [GGQAMHO]^+^ (calcd. for C_22_H_37_N_4_O_8_^+^, 485.2606) (7) 430.2043 [GGGQHO]^+^ (calcd. for C_16_H_28_N_7_O_7_^+^, 430.2045) (19). For other data see Fig. 6 which is the average of the spectra obtained by HRMS^2^ (CID @ 35% CE, HCD @ 10, 15, 20, 25, 35, 40 and 45 eV).

### 3.11 Epichloëamide (**3**)

Analytical HPLC: Rt = 14.32 min (gradient 1): HRMS (positive ESI) *m*/*z*: 518.2792 [M+2H]^2+^ (calcd. for C_46_H_76_N_12_O_15_^2+^, 518.2771), HRMS^2^ (CID) 518.3 @ 35% CE, *m*/*z* 923.4945 [M+H−AM]^+^ (calcd. for C_40_H_67_N_12_O_13_^+^, 923.4945) (100), *m*/*z* 921.4678, [M+H−Orn]^+^ (calcd. for C_41_H_65_N_10_O_14_^+^, 921.4676) (5), 509.2741 [M+2H−H_2_O]^2+^ (calcd. for C_46_H_74_N_12_O_14_^++^, 509.2718) (90) 462.2531 [M+2H−AM]^2+^ (calcd. for C_40_H_68_N_12_O_13_^++^, 462.2509) (55). HRMS^3^ (CID) 518.3@35% CE; 923 @ 35% CE: 905.4847 [M+H−AM−H_2_O] (calcd. for C_40_H_65_N_12_O_12_^+^, 905.4839) (56), 809.4165 [GGGGQ(AMO)_2_]^+^ (calcd. for C_35_H_57_N_10_O_12_^+^, 809.4152) (100), 791.4059 [GGGGQ(AMO)_2_−H_2_O]^+^ (calcd. for C_35_H_55_N_10_O_11_^+^, 791.4046) (14), 697.3638 [GGGGQ(AMO)O]^+^ (calcd. for C_29_H_49_N_10_O_10_^+^, 697.3628) (12), 640.3423 [GGGQ(AMO)O]^+^ (calcd. for C_27_H_46_N_9_O_9_^+^, 640.3413) (5), 624.3367 [GGG(AMO)_2_]^+^ (calcd. for C_28_H_46_N_7_O_9_^+^, 624.3351) (1.5), 583.2847 [GGGGQ(AMO)]^+^ (calcd. for C_24_H_39_N_8_O_9_^+^, 583.2835) (12), 567.3518 [(AMO)_2_O]^+^ (calcd. for C_27_H_47_N_6_O_7_^+^, 567.3501) (4), 471.2323 [GGGGQO]^+^ (calcd. for C_18_H_31_N_8_O_7_^+^, 471.2310) (12). For additional data see Fig. 7 which is the average of the spectra obtained by HRMS^3^ (518.3 CID @ 35% CE; 923.4 (CID @ 35% CE, HCD @ 10, 15, 20, 25, 35, 40 and 45 eV)).

## Footnotes

1 Calculated and observed *m/z* values for these ions were generally consistent within 5 ppm, and alternative formulae could be excluded as inconsistent.

2 For many of these MS^3^ data the differential between observed and calculated *m/z* values exceeded 5 ppm, but alternative formulae with similar *m/z* differentials could be excluded as inconsistent.
