# Supplementary material for: Identification of extracellular siderophores and a related peptide from the endophytic fungus Epichloë festucae in culture and endophyte-infected Lolium perenne
Source: Phytochemistry. 2012 Mar;75(6):128–39. doi: 10.1016/j.phytochem.2011.11.020 (PMC3311397; doi:10.1016/j.phytochem.2011.11.020)

**Supporting information**

1. ^1^H NMR of epichloënin A (**1**) at 600 MHz (DMSO-*d_6_*)

2. ^13^C NMR of epichloënin A (**1**) at 150 MHz (DMSO-*d_6_*)

3. ^1^H-^1^H COSY NMR of epichloënin A (**1**) at 600 MHz (DMSO-*d_6_*)

4. ^1^H-^13^C edited HSQC NMR of epichloënin A (**1**) at 600 MHz and 150 MHz (DMSO-*d_6_*)

5. ^1^H-^13^C HMBC NMR of epichloënin A (**1**) at 600 MHz and 150 MHz (DMSO-*d_6_*)

6. ^1^H-^1^H NOESY NMR of epichloënin A (**1**) at 600 MHz (DMSO-*d_6_*)

6a. Expansion of the ^1^H-^1^H NOESY NMR of epichloënin A (**1**) at 600 MHz (DMSO-*d_6_*) showing NOEs between the olefinic and allylic protons of the *trans-*AMHO moieties

7. CD spectrum of ferriepichloënin A (**1-Fe**)

1. ^1^H NMR of epichloënin A (**1**) at 600 MHz (DMSO-*d_6_*)


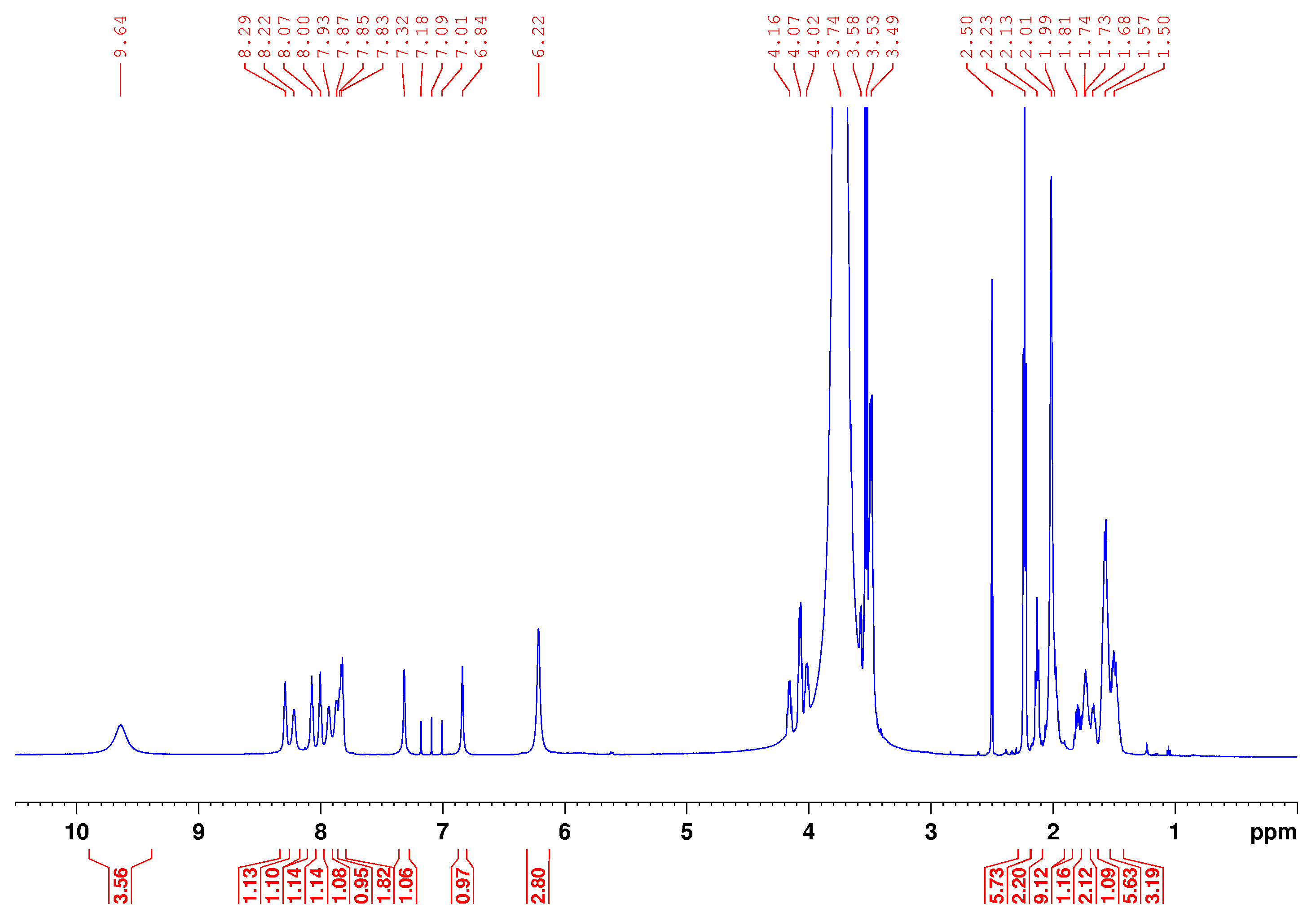


2. ^13^C NMR of epichloënin A (**1**) at 150 MHz (DMSO-*d_6_*)


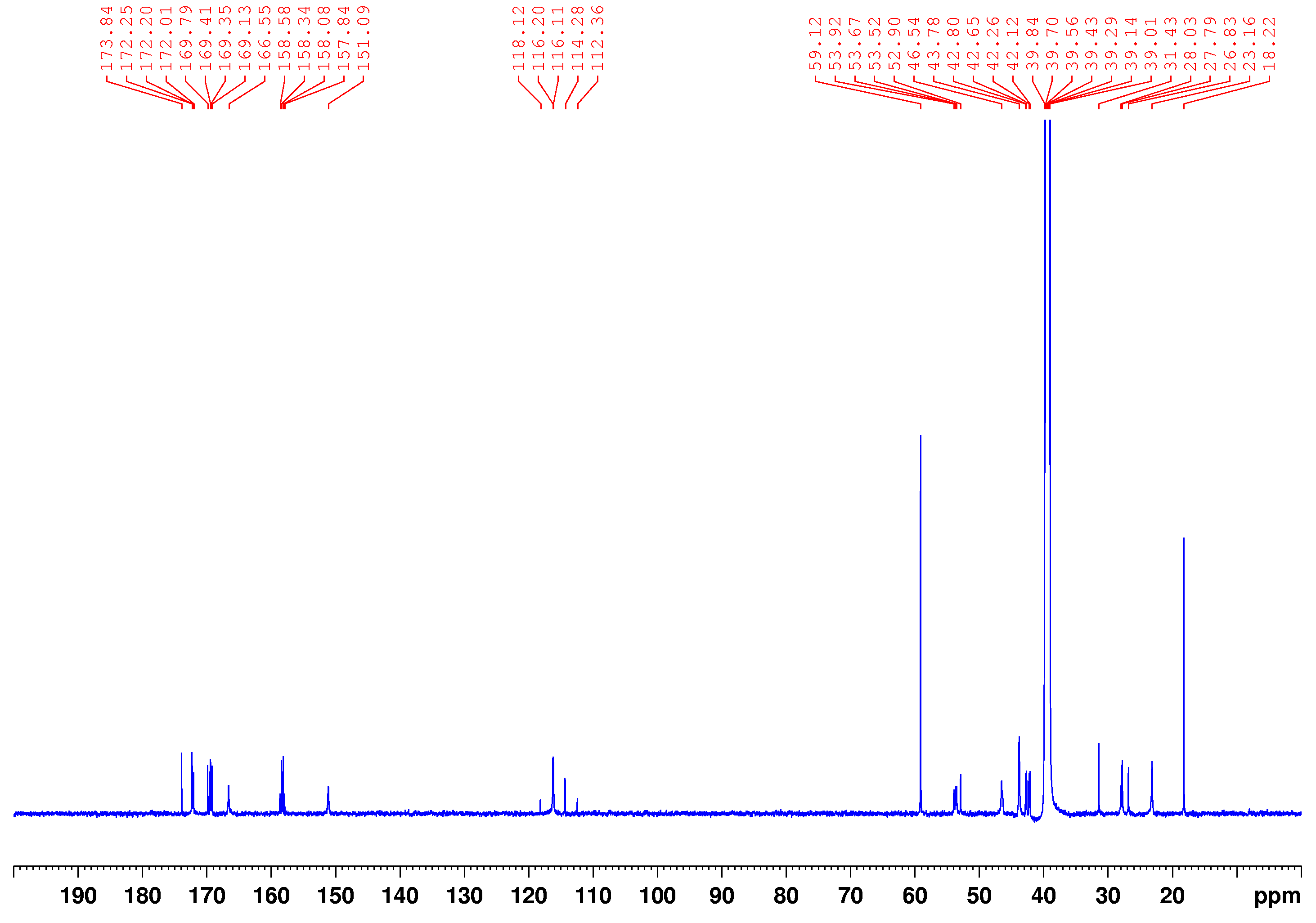


3. ^1^H-^1^H COSY NMR of epichloënin A (**1**) at 600 MHz (DMSO-*d_6_*)


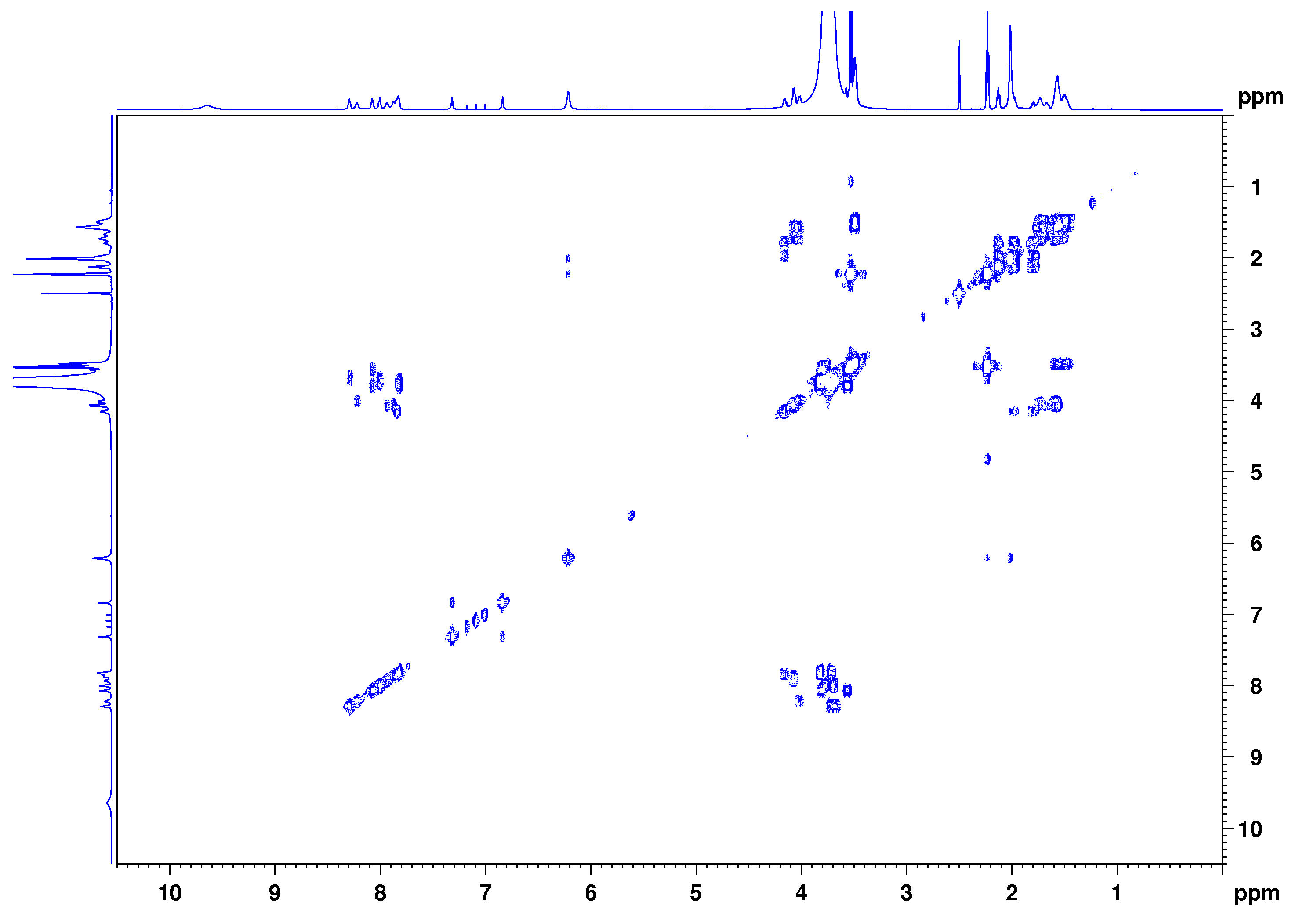


4. ^1^H-^13^C edited HSQC NMR of epichloënin A (**1**) at 600 MHz and 150 MHz (DMSO-*d_6_*)


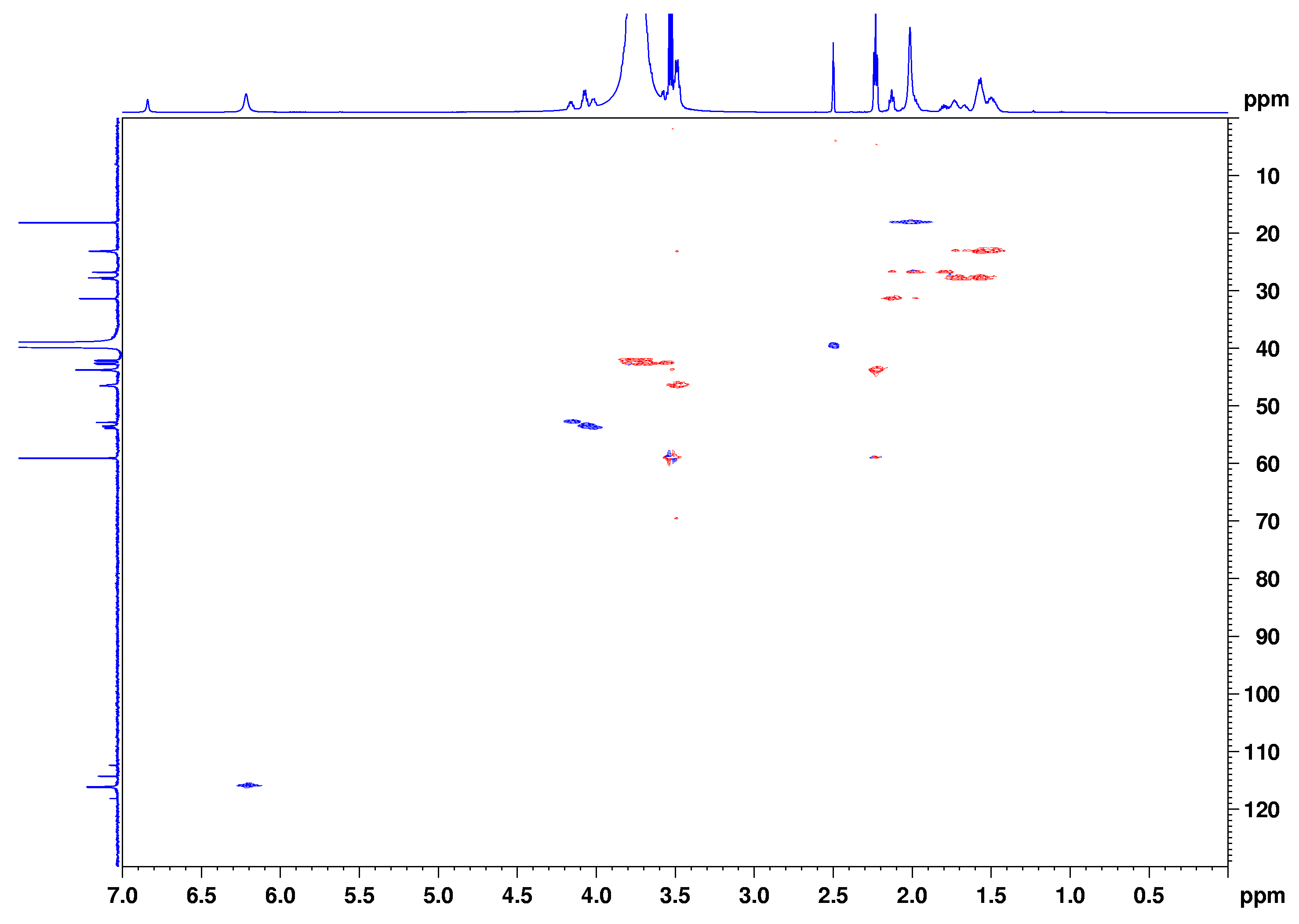


5. ^1^H-^13^C HMBC NMR of epichloënin A (**1**) at 600 MHz and 150 MHz (DMSO-*d_6_*)


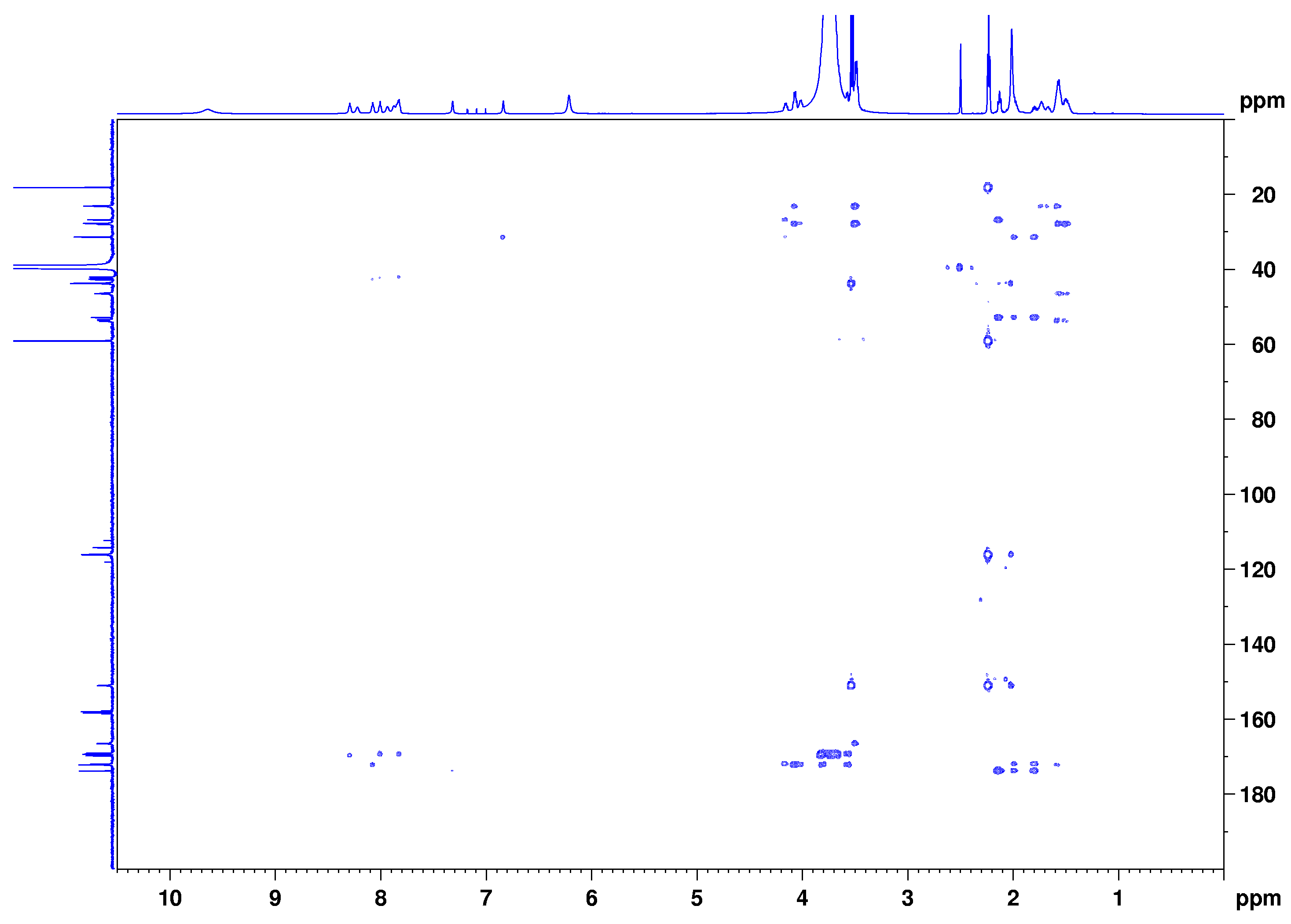


6. ^1^H-^1^H NOESY NMR of epichloënin A (**1**) at 600 MHz (DMSO-*d_6_*)


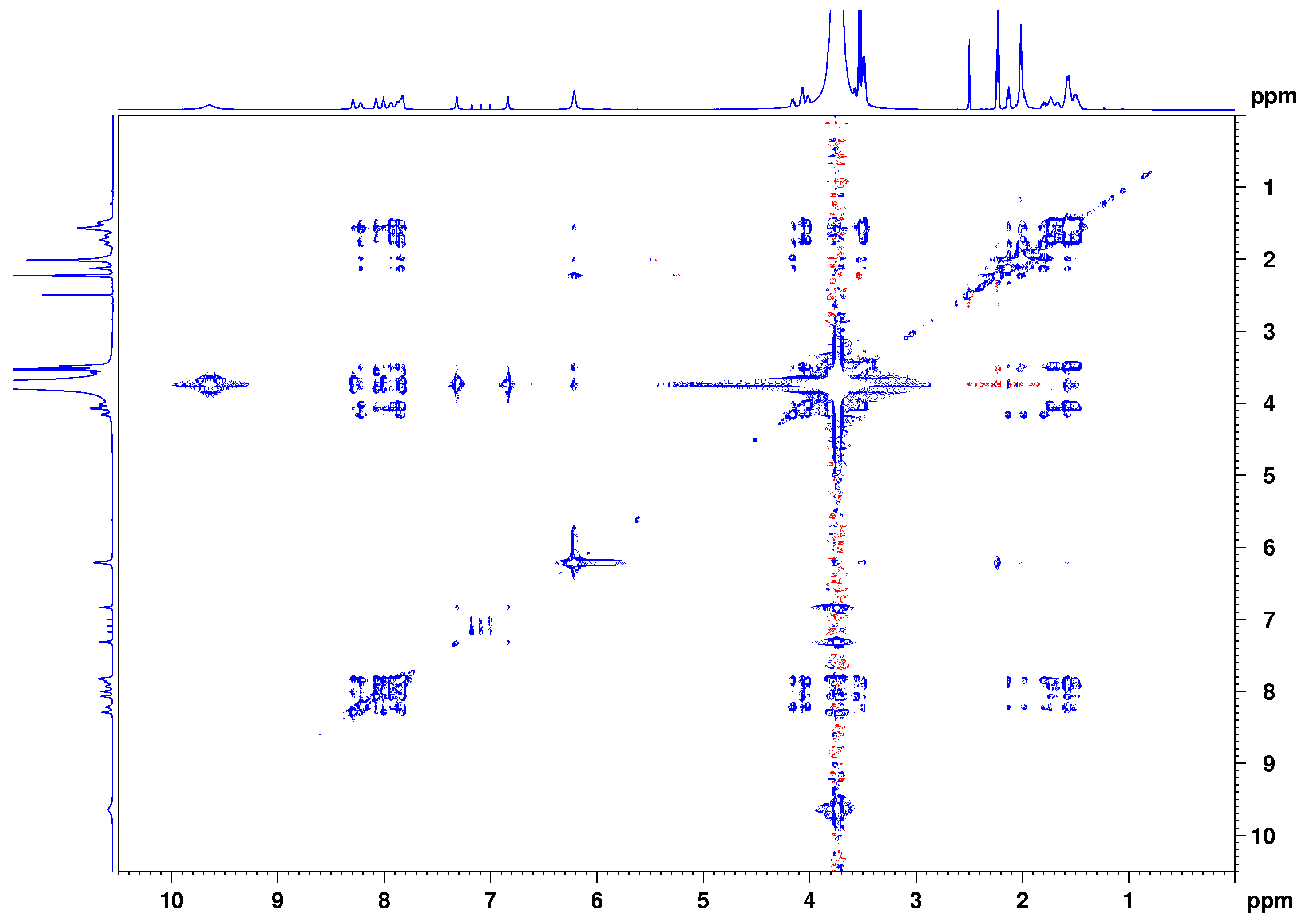


6a. Expansion of the ^1^H-^1^H NOESY NMR of epichloënin A (**1**) at 600 MHz (DMSO-*d_6_*) showing NOEs between the olefinic and allylic protons of the *trans-*AMHO moieties.


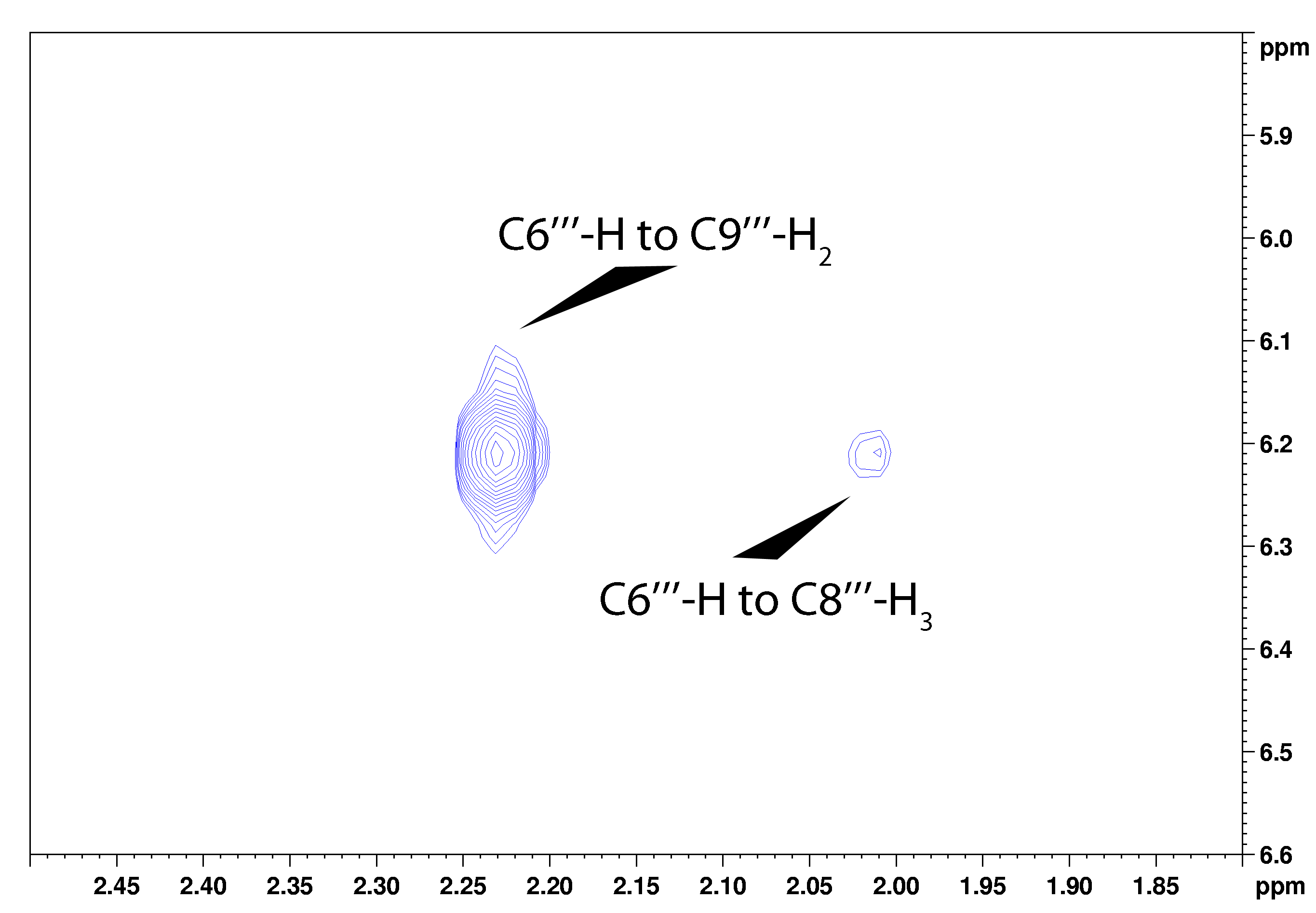


7. CD spectrum of ferriepichloënin A (**1-Fe**)


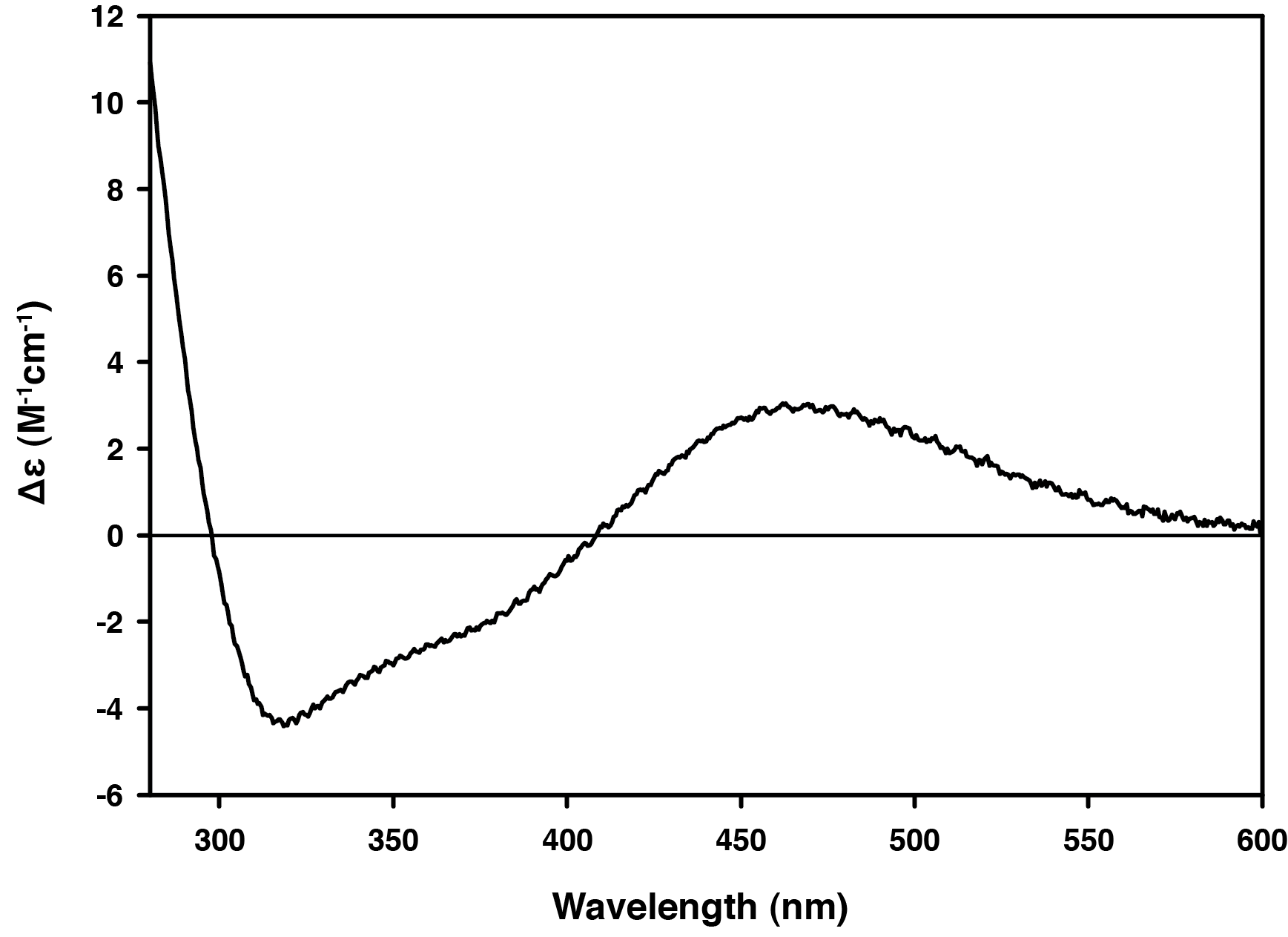

Supplement: Supplementary data 2 [file mmc1.doc]
